# Employment-dependent associations of serum biomarkers with short- and long-term antidepressant treatment outcomes

**DOI:** 10.3389/fpsyt.2025.1662993

**Published:** 2026-01-09

**Authors:** Jae-Min Kim, Hee-Ju Kang, Ju-Wan Kim, Min Jhon, Ju-Yeon Lee, Sung-Wan Kim, Il-Seon Shin

**Affiliations:** Department of Psychiatry, Chonnam National University Medical School, Gwangju, Republic of Korea

**Keywords:** depression, employment, pharmacotherapy, remission, serum biomarker

## Abstract

**Background:**

This study investigated whether employment status moderates associations between baseline serum biomarkers and antidepressant remission at 12 weeks and 12 months.

**Methods:**

A prospective cohort of 1086 outpatients diagnosed with depressive disorders received stepwise antidepressant therapy using a naturalistic, flexible treatment protocol. Fourteen serum biomarkers covering immune (hsCRP, TNF-α, IL-1β, IL-6, IL-4, IL-10), metabolic (leptin, ghrelin, total cholesterol), neuroplastic (BDNF), neurotransmitter (serotonin), endocrine (cortisol), and nutritional (folate, homocysteine) domains were analyzed at baseline. Employment-dependent biomarker associations with remission (Hamilton Depression Rating Scale ≤7) at 12 weeks and 12 months were evaluated using logistic regression with biomarker-by-employment interactions and stratified analyses, adjusting for relevant covariates.

**Results:**

Higher serotonin levels significantly predicted 12-week remission exclusively among employed patients, with a significant employment interaction. At 12 months, lower leptin levels predicted remission specifically in employed patients, whereas lower TNF-α and higher BDNF levels predicted remission only in unemployed patients, each demonstrating significant employment interactions.

**Conclusion:**

Baseline serum biomarkers showed employment-dependent associations with antidepressant remission outcomes, highlighting serotonin’s short-term relevance and leptin, TNF-α, and BDNF as longer-term indicators. Although exploratory, these findings suggest that integrating employment status with biomarker profiles may enhance clinical decision-making by identifying patients who are more or less likely to benefit from treatment across different phases of recovery. Replication in independent cohorts is needed to establish the clinical applicability of such employment-tailored, biomarker-informed strategies.

## 1 Introduction

Employment status is a significant social determinant of depression. Large population studies show that being employed, particularly full-time, is associated with substantially lower depression prevalence compared to unemployment or economic inactivity (1). Conversely, job loss and precarious work often precede increases in depressive symptoms and poorer clinical outcomes. Unemployment imposes financial strain and psychosocial stress, heightening vulnerability to depression (2). These observations suggest that employment provides stability and social support, buffering against depression, whereas unemployment-induced stress elevates risk and may worsen illness progression. Indeed, employed individuals generally achieve better recovery and remission rates from depression treatment compared to their unemployed counterparts (3), emphasizing how socioeconomic context influences depression outcomes.

Employment status also affects depression-related biological markers. Chronic stress associated with unemployment activates inflammatory pathways; unemployed individuals often exhibit elevated peripheral inflammatory markers such as high-sensitivity C-reactive protein (hsCRP) and interleukin-6 (IL-6) compared to employed individuals (4). Such immune activation contributes to depression pathophysiology. Low socioeconomic status has similarly been linked to increased inflammation and reduced brain-derived neurotrophic factor (BDNF), potentially impairing neuroplasticity (5). Additionally, unemployment represents a potent chronic stressor that may lead to sustained hyperactivation of the hypothalamic–pituitary–adrenal (HPA) axis (2), subsequently disrupting metabolic and neurotransmitter systems. Metabolic consequences include higher obesity and insulin resistance risks (2), linked to depression via an immuno-metabolic pathway involving inflammation and oxidative stress. Furthermore, inflammatory cytokines induced by chronic stress can redirect tryptophan metabolism away from serotonin synthesis toward the neurotoxic kynurenine pathway, reducing central serotonin availability and exacerbating depressive symptoms (6). Nutritional factors also play a role; economic hardship may impair diet quality, leading to deficiencies in essential nutrients such as folate, which directly influence neurotransmitter synthesis and inflammatory processes (7). Thus, employment status can influence various depression-related biomarkers, providing a mechanistic link between unemployment-induced stress and depressive pathology.

Although employment clearly affects depression outcomes and associated biomarkers, it remains uncertain whether employment status moderates biomarker-based predictions of treatment response in depression. Specifically, do biological indicators of treatment prognosis differ in predictive strength between employed and unemployed patients? To date, most biomarker studies examining depression treatment outcomes have not considered social determinants like employment as interacting variables. This gap highlights the need for integrated approaches incorporating both biological and social factors in predicting treatment responses.

Given the influence of employment status on depression and its associated biomarkers, we propose that employment status may interact with biomarkers to affect treatment prognosis. Employment provides psychosocial stability that might mitigate the adverse impact of certain biomarker abnormalities, while unemployment could exacerbate biological vulnerabilities. In a prior study using the same prospective cohort, we developed a multimodal serum biomarker panel—comprising 14 biomarkers across multiple biological systems—that significantly predicted 12-week and 12-month remission outcomes in depressive disorders (8). Building on this work, we hypothesize that employment status moderates the relationship between baseline biomarkers and treatment response. Because the present study aims to identify preliminary patterns rather than test predefined hypotheses, our design and analytic strategy intentionally follow an exploratory, hypothesis-generating framework rather than a confirmatory one. Demonstrating such interactions would emphasize the importance of integrating social determinants into biomarker-informed depression care, ultimately advancing personalized and equitable treatment approaches.

## 2 Methods

This study is part of the MAKE Biomarker discovery for Enhancing Antidepressant Treatment Effect and Response (MAKE BETTER) program, designed to explore clinically relevant biomarkers influencing antidepressant treatment outcomes. Comprehensive methodological details for this broader initiative have been documented elsewhere (9). In brief, the present analysis involved patients diagnosed with depressive disorders, representing diverse clinical characteristics typically encountered in routine practice. At baseline, participants underwent detailed assessments capturing sociodemographic and clinical variables, with a particular focus on employment status, alongside blood sampling for biomarker quantification. Following initial assessments, patients received antidepressant monotherapy, which was systematically reviewed and adjusted according to structured remission evaluations conducted every three weeks for the initial 12-week period, and subsequently every three months up to one year. Individual treatment plans and subsequent modifications were informed by patient-specific clinical presentations and preferences, following established clinical guidelines previously reported (10). Data collection procedures employed structured case report forms completed by research coordinators who remained blind to clinical management decisions to ensure unbiased recording and confidentiality. The study protocol was ethically approved by the Institutional Review Board of Chonnam National University Hospital (CNUH 2012-014).

### 2.1 Participants

Participants were recruited from outpatient psychiatric services at CNUH from March 2012 to April 2017. Inclusion criteria encompassed adults aged ≥18 years diagnosed with major depressive disorder, dysthymic disorder, or depressive disorder not otherwise specified according to DSM-IV-TR (11), and having a baseline Hamilton Depression Rating Scale (HAMD) score ≥14 (12). Key exclusion criteria included severe or unstable medical conditions, conditions impairing reliable psychiatric assessment or medication adherence, history of bipolar disorder, schizophrenia, other severe psychotic or organic mental disorders, seizure disorders, recent psychiatric hospitalization unrelated to depression, recent electroconvulsive therapy, and pregnancy or breastfeeding. Additionally, patients who had recently received antidepressant treatment were excluded, ensuring participants were initiating antidepressant therapy for either a first or recurrent depressive episode. All patients provided written informed consent prior to enrollment.

### 2.2 Baseline characteristics

#### 2.2.1 Employment status

In this study, employment status was defined dichotomously by the presence or absence of any personal income (e.g., wages from employment or earnings from a business). Participants reporting any annual income were categorized as “employed,” whereas those with no reported income – including full-time homemakers – were classified as “unemployed”. This operationalization mirrors methodologies in socioeconomic and psychiatric research, where individuals are commonly grouped simply as employed versus non-employed to assess the influence of economic activity on mental health outcomes and psychosocial well-being (1, 13).

#### 2.2.2 Serum biomarkers

Participants were instructed to fast overnight and remain at rest for approximately 30 minutes prior to morning blood collection. Venous blood (10 mL) was drawn into serum-separating tubes, stored at 2–4 °C for several hours, then centrifuged at 3000×g for 15 minutes at 4 °C. Resulting serum samples were aliquoted and stored at −80 °C until assay. To minimize pre-analytical variability, samples were thawed only once immediately before testing. All biomarker assays were conducted at the Global Clinical Central Lab (Yongin, Korea), with laboratory staff blinded to participants’ clinical information.

Fourteen serum biomarkers spanning six biological domains were selected based on prior meta-analytic and empirical evidence (14). Immune markers included hsCRP (Tina-quant assay, Roche), TNF-α (Quantikine HS ELISA, R&D Systems), and cytokines IL-1β, IL-6, IL-4, and IL-10 (High Sensitivity T Cell Magnetic Bead Panel, EMD Millipore). Metabolic markers included leptin (BioVendor ELISA), total ghrelin (EMD Millipore radioimmunoassay), and total cholesterol (Wako enzymatic method). Neuroplasticity was indexed by BDNF (Quantikine ELISA, R&D Systems). Serotonin, representing the neurotransmitter domain, was measured via high-performance liquid chromatography (ClinRep, Recipe). Cortisol, the endocrine marker, was analyzed using the Cobas Cortisol II electrochemiluminescence immunoassay (Roche). Nutritional status was assessed via folate (Cobas Elecsys Folate III, Roche) and homocysteine (ARCHITECT 1L71, Abbott) assays. Data on inter-assay coefficients of variation of the biomarkers are summarized in **Supplementary Table S1**.

#### 2.2.3 Socio-demographic and clinical characteristics

Comprehensive socio-demographic information was obtained at baseline, including participants’ age, sex, years of formal education, marital status (married vs. unmarried), living arrangement (living alone vs. with others), religious affiliation (yes/no), and self-reported household monthly income (dichotomized at 2,000 USD). Because this variable reflects total household income rather than the participant’s personal earnings, full-time homemakers could report higher household income while being classified as unemployed. Clinical characteristics were assessed through structured interviews and included DSM-IV-TR depressive disorder subtype and specifiers, age at first onset, duration and recurrence of depressive episodes, current episode duration, family history of depression, and number of coexisting physical illnesses based on a 15-system medical checklist. Additional baseline variables included body mass index (BMI) and smoking status. Psychiatric symptom severity and functional impairment were evaluated using validated rating scales: the Hospital Anxiety and Depression Scale (HADS) subscales for depression (HADS-D) and anxiety (HADS-A) (15), the Social and Occupational Functioning Assessment Scale (SOFAS) (11), and the Alcohol Use Disorder Identification Test (AUDIT) (16). Higher scores on HADS-D, HADS-A, and AUDIT reflected greater symptom burden, whereas lower SOFAS scores indicated greater functional impairment.

### 2.3 Stepwise pharmacotherapy

The stepwise pharmacotherapy protocol used in this study has been described in detail elsewhere [10]. Prior to treatment initiation, participants received a structured clinical assessment covering depressive symptom severity, medical comorbidities, concomitant medications, and prior antidepressant treatment history when applicable. Initial antidepressant monotherapy was selected according to standardized clinical guidelines (17), tailored to each patient’s presentation. Treatment progress was reviewed every three weeks during the acute phase. If symptom improvement was insufficient or adverse effects emerged, treatment adjustments followed a flexible, algorithm-guided framework. Modifications included switching to a different antidepressant, augmenting with an additional agent, or combining antidepressants, as well as various combinations of these strategies. All treatment decisions incorporated patient preferences and clinical judgment, ensuring individualized care within evidence-based parameters.

### 2.4 Definition of remission

Remission was evaluated at multiple time points during both the acute (weeks 3, 6, 9, and 12) and continuation (months 6, 9, and 12) phases of treatment. Remission was operationally defined as a Hamilton Depression Rating Scale (HAMD) score ≤7 at each time point. For the 12-week remission analysis, participants were included if they had undergone at least one follow-up assessment within the first 12 weeks. The 12-month remission analysis included those who completed at least one evaluation during both the acute (within 12 weeks) and continuation (6–12 months) periods. In both cases, remission status was confirmed only if maintained through the final assessment at week 12 or month 12, respectively.

### 2.5 Statistical analysis

Baseline socio-demographic and clinical characteristics were compared according to employment and remission status at 12 weeks and 12 months using independent t-tests or chi-square (χ²) tests. Variables associated significantly (P < 0.05) with either employment or remission outcomes, or identified as relevant from previous literature (18), were selected as candidate covariates after assessing for potential multicollinearity. Serum biomarker levels were compared between employed and unemployed participants using Mann-Whitney U tests. Biomarker analyses were conducted using both categorical and continuous approaches. For categorical analyses, biomarkers were dichotomized at median values to enhance statistical robustness and balanced subgroup comparisons. The choice of median cut-off and expected directionality of biomarker associations were guided by previous findings (8, 19). Associations between these categorical biomarker variables and remission outcomes were evaluated using binary logistic regression models, adjusted for covariates. To investigate whether employment status moderated these associations, analyses were stratified by employment group, and interaction terms between biomarkers and employment status were tested using multinomial logistic regression models with similar covariate adjustments. For continuous analyses, biomarkers were evaluated using logistic regression to explore linear, dose-response relationships, again considering potential moderation by employment status through interaction terms. Because logistic regression does not require predictors to be normally distributed, biomarkers were analyzed on their original scales without log-transformation. All statistical tests were two-sided, with significance set at α = 0.05. Given the exploratory nature of this analysis intended for hypothesis generation, corrections for multiple comparisons (such as Bonferroni adjustments) were not applied to prevent overly conservative interpretations. This approach was chosen to preserve sensitivity to potential biomarker patterns that may warrant further study, rather than to make confirmatory inferential claims based on adjusted significance thresholds. All analyses were conducted using complete-case data; no imputation procedures were applied. Statistical analyses were performed using SPSS version 21.0 (IBM Corp., Armonk, NY, USA).

## 3 Results

### 3.1 Recruitment

**Supplementary Figure S1** illustrates participant recruitment and flow throughout the study. Of 1262 patients initially screened, serum samples were successfully collected from 1094 (86.7%). Among these, 1086 patients (86.1%) completed at least one follow-up assessment within the initial 12-week treatment period. Baseline demographic and clinical characteristics were comparable between the 1086 patients included and the 176 patients excluded from analyses (all P > 0.1). During the continuation phase up to 12 months, 884 (81.4%) of the initial 1086 participants remained engaged, completing at least one additional evaluation. Attrition by the 12-month assessment was significantly related to unemployment and the presence of melancholic features at baseline. No other baseline socio-demographic or clinical characteristics differed significantly between participants who completed the 12-month follow-up and those who did not attend follow-up assessments, indicating that attrition was selectively associated with these two factors only. For transparency, the analytic sample sizes at each phase were: 1262 screened, 1094 with serum samples, 1086 contributing to 12-week analyses, and 884 contributing to 12-month analyses (**Supplementary Figure S1**).

### 3.2 Baseline characteristics by employment and remission status

Among the 1086 participants evaluated at least once during the initial 12-week treatment phase, 316 (29.1%) were unemployed. Baseline characteristics stratified by employment status are summarized in **Table 1**. Compared to employed participants, those unemployed were significantly more likely to be older, male, unmarried, living alone, and to have lower monthly income, older age at depression onset, more depressive episodes, greater depressive symptom severity (higher HADS-D scores), and poorer functional status (lower SOFAS scores). Remission was achieved by 490 (45.1%) participants at 12 weeks and by 625 (70.7%) participants at the subsequent 12-month follow-up. Baseline characteristics by remission status at these intervals are presented in **Supplementary Table S2**. Remission at 12 weeks was significantly associated with older age, higher monthly income, older age at depression onset, fewer previous depressive episodes, shorter current episode duration, non-smoking status, lower depression and anxiety symptom severity (lower HADS-D and HADS-A scores), and better functional status (higher SOFAS scores). Remission at 12 months was associated significantly with higher monthly income, fewer depressive episodes, and lower scores on both HADS-D and HADS-A. Based on these observed associations, prior literature (18), and absence of multicollinearity, nine covariates were included in adjusted analyses: age, sex, marital status, monthly income, number of depressive episodes, number of physical disorders, BMI, HADS-D, and SOFAS scores. Baseline serum biomarker levels stratified by employment status are displayed in **Table 2**. Unemployed participants exhibited significantly lower folate and higher homocysteine levels.

**Table 1:** Baseline characteristics by employment status (N = 1086).

Variables	Employed (N = 770)	Unemployed (N = 316)	Statistical coefficients^a^	P-value
Socio-demographic characteristics				
Age, mean (SD) years	56.2 (14.02)	58.8 (16.7)	t=-2.461	**0.014**
Sex, N (%) female	552 (71.7)	192 (61.1)	χ^2^ = 11.714	**0.001**
Education, mean (SD) years	9.2 (4.9)	8.9 (4.6)	t=+0.884	0.377
Marital status, N (%) unmarried	193 (25.1)	133 (42.1)	χ^2^ = 30.909	**<0.001**
Living alone, N (%)	95 (12.3)	72 (22.8)	χ^2^ = 18.792	**<0.001**
Household monthly income, N (%) <2,000 USD	416 (54.0)	232 (73.4)	χ^2^ = 35.010	**<0.001**
Clinical characteristics				
Major depressive disorder, N (%)	653 (84.8)	272 (86.1)	χ^2^ = 0.287	0.592
Melancholic feature, N (%)	127 (14.5)	50 (15.8)	χ^2^ = 0.288	0.591
Atypical feature, N (%))	50 (6.5)	19 (6.0)	χ^2^ = 0.087	0.768
Age at onset, mean (SD) years	50.0 (15.9)	53.3 (17.9)	t=-2.816	**0.005**
Duration of illness, mean (SD) years	6.5 (9.5)	5.8 (8.9)	t=+1.012	0.312
Number of depressive episodes, mean (SD)	2.0 (4.5)	2.9 (6.3)	t=-2.299	**0.022**
Duration of present episode, mean (SD) months	7.3 (10.2)	7.6 (10.8)	t=-0.492	0.623
Family history of depression, N (%)	119 (15.5)	39 (12.3)	χ^2^ = 1.746	0.186
Number of physical disorders, mean (SD)	1.6 (1.3)	1.7 (1.3)	t=-0.921	0.357
Body mass index, mean (SD) kg/m^2^	23.2 (3.2)	23.2 (3.2)	t=+0.043	0.966
Current smoking, N (%)	81 (10.5)	42 (13.3)	χ^2^ = 1.714	0.190
Assessment scales, mean (SD) scores				
Hospital Anxiety & Depression Scale-depression subscale	13.4 (4.0)	14.4 (3.7)	t=-3.946	**<0.001**
Hospital Anxiety & Depression Scale-anxiety subscale	11.7 (4.0)	12.1 (4.0)	t=-1.477	0.140
Social and Occupational Functional Assessment Scale	56.8 (7.3)	53.8 (7.5)	t=+6.160	**<0.001**
Alcohol Use Disorders Identification Test	5.6 (8.9)	4.9 (8.8)	t=+1.148	0.251

a Independent two sample t-test or χ^2^ tests, as appropriate.

Bold style denotes statistical significance (p-values<0.05).

**Table 2:** Baseline median (interquartile range) levels of serum biomarkers by employment status (N = 1086).

Serum biomarker	Employed (N = 770)	Unemployed (N = 316)	U-value^a^	P-value
High-sensitivity C-reactive protein, mg/L	0.4 (1.0)	0.5 (0.9)	127806.5	0.188
Tumor necrosis factor-α, pg/mL	0.6 (0.4)	0.6 (0.4)	123428.5	0.706
Interleukin-1β, pg/mL	1.1 (0.8)	1.1 (0.8)	127257.0	0.233
Interleukin-6, pg/mL	1.6 (1.6)	1.7 (1.6)	122729.0	0.820
Interleukin-4, pg/mL	36.8 (37.6)	37.3 (39.5)	126274.0	0.326
Interleukin-10, pg/mL	10.5 (9.8)	10.9 (9.6)	124685.5	0.519
Leptin, ng/mL	5.7 (6.1)	6.0 (6.7)	124750.0	0.510
Ghrelin, pg/mL	382.0 (185.3)	376.0 (170.8)	117941.5	0.428
Total cholesterol, mg/dL	178.0 (53.3)	176.5 (53.0)	119390.5	0.629
Brain derived neurotrophic factor, ng/mL	23.2 (8.7)	23.4 (8.5)	118773.5	0.539
Serotonin, ng/mL	74.0 (64.6)	68.9 (75.4)	117622.5	0.390
Cortisol, μg/dL	10.6 (5.5)	10.9 (6.2)	126988.5	0.256
Folate, ng/mL	7.8 (5.9)	7.0 (5.8)	113529.0	**0.033**
Homocysteine, μmol/L	10.7 (4.5)	11.7 (5.1)	140699.0	**<0.001**

a Mann-Whitney U tests.

Bold style denotes statistical significance (p-values<0.05).

### 3.3 Associations between categorical serum biomarkers and remission status

**Figure 1:**
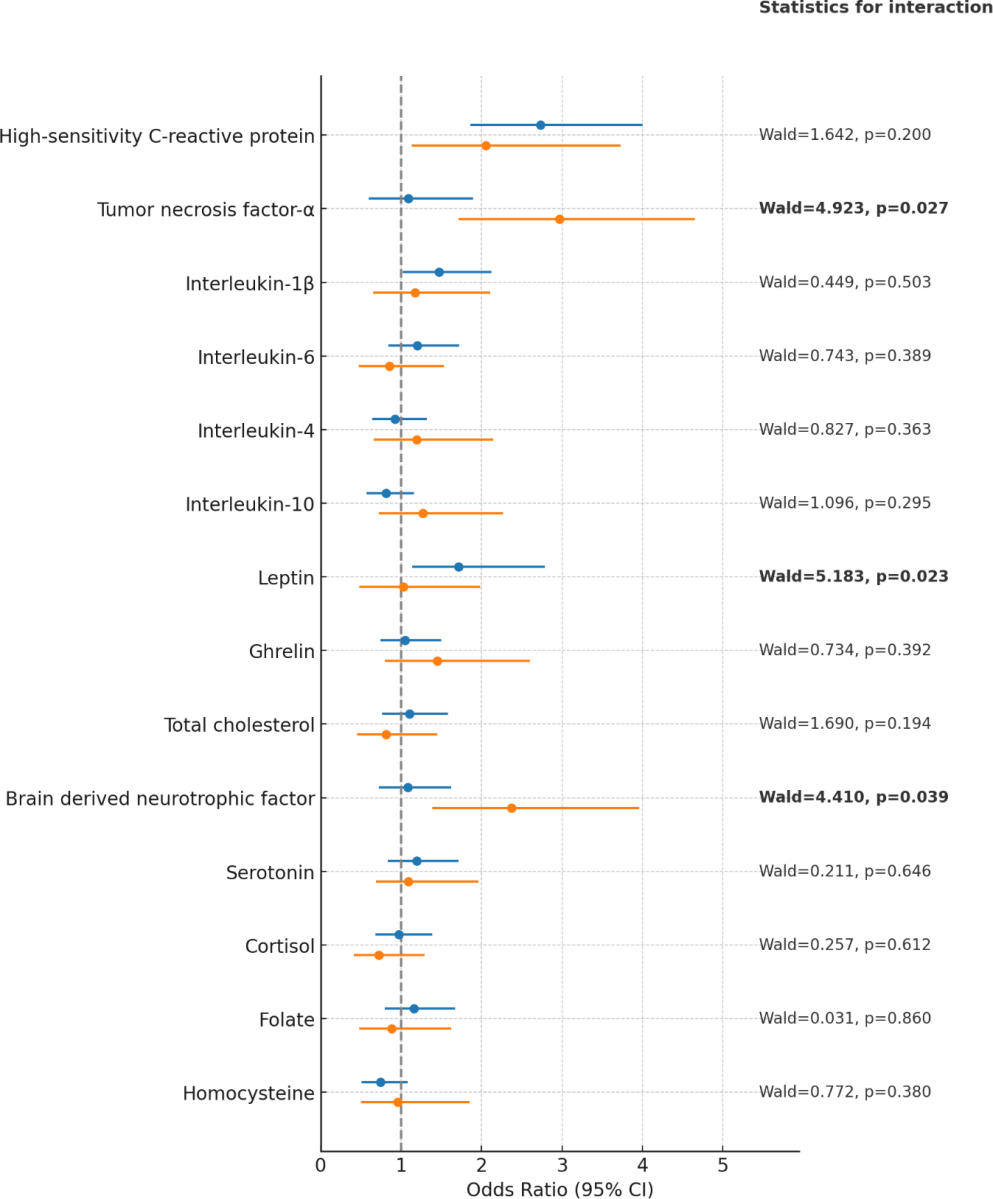
Forest plot of adjusted odds ratios for 12-month remission by biomarker, stratified by employment status, with interaction statistics. Odds ratios and 95% confidence intervals (95% CIs) are estimated from logistic regression models adjusted for age, sex, marital status, monthly income, number of depressive episodes, number of physical disorders, body mass index, and scores on the Hospital Anxiety & Depression Scale–Depression Subscale and the Social and Occupational Functioning Assessment Scale. Blue markers indicate the employed group and orange markers indicate the unemployed group. Bolded statistics denote biomarker-by-employment interactions with p < 0.05.

**Supplementary Table S3** summarizes the associations between median-dichotomized serum biomarkers and 12-week remission. Several biomarkers showed significant associations within employment strata; however, no employment-by-biomarker interactions reached statistical significance at this timepoint. For 12-month outcomes, a visual summary is provided in **Figure 1**, and full estimates appear in **Supplementary Table S4**. At 12 months, significant employment-dependent interactions were observed for TNF-α, leptin, and BDNF, with remission associated with lower TNF-α and higher BDNF among unemployed participants and with lower leptin among employed participants. No other biomarkers demonstrated significant interaction effects with employment status.

### 3.4 Dose-dependent employment interactions between continuous serum biomarkers and remission status

To further explore potential dose-dependent relationships underlying the previously described categorical biomarker interactions, serum biomarkers were analyzed as continuous variables concerning 12-week and 12-month remission outcomes, with employment-dependent interactions summarized in **Supplementary Table S5**. Regarding 12-week remission, serotonin—which did not exhibit a significant interaction in the categorical analyses—showed a statistically significant continuous interaction, indicating that higher serotonin levels were continuously and positively associated with remission specifically among employed participants. For 12-month remission outcomes, results among unemployed participants aligned consistently with the categorical analyses. Specifically, statistically significant employment status-by-biomarker interactions were observed for continuous measures of TNF-α, leptin, and BDNF after adjustment for all covariates.

## 4 Discussion

In this prospective study of patients with depressive disorders undergoing stepwise pharmacological therapy, employment-dependent associations between baseline serum biomarkers and antidepressant remission outcomes were identified. Higher serotonin levels were significantly associated with remission at 12 weeks exclusively among employed patients. At 12 months, lower leptin levels were associated with remission specifically in employed patients, whereas lower TNF-α and higher BDNF levels were significantly associated with remission exclusively among unemployed individuals.

Several methodological considerations should be noted when interpreting these findings. Prior biomarker studies often employed tightly controlled randomized trial designs examining limited antidepressant options (20). In contrast, our study used a flexible, sequential pharmacotherapy protocol with regular evaluations and adjustments every three weeks, reflecting patient preferences, efficacy, and tolerability. Although this pragmatic approach enhances external validity, it introduces variability in treatment regimens and outcomes. Furthermore, unemployed participants had higher attrition rates at 12 months, associated with unfavorable social and clinical characteristics—such as lower income, social isolation, higher depressive severity, and poorer functional status—which might have reduced rather than exaggerated observed associations. Nonetheless, differential attrition remains an important potential source of bias, and the direction and magnitude of its impact cannot be fully determined. Future studies with more intensive retention efforts or formal weighting/imputation approaches will be needed to validate these findings. Lastly, several employment-by-biomarker interactions reached nominal statistical significance but would not survive multiple comparison corrections. Given the exploratory nature of the analysis, these findings should be cautiously interpreted and viewed as hypothesis-generating until replicated in future research. While more stringent confirmatory procedures (e.g., multiplicity correction, outcome redefinition, advanced weighting methods) are valuable in hypothesis-testing designs, they are not fully compatible with the exploratory objectives and naturalistic clinical framework of the present study.

Higher baseline serotonin appeared to show an association consistent with an acute treatment advantage specifically among employed patients, indicating a potential synergy between biological resilience and favorable psychosocial conditions in early antidepressant response. Biologically, serotonin acts as a stress-buffering neurotransmitter, facilitating mood stabilization during acute recovery phases (21). Psychosocially, employed individuals benefit from structured routines, social engagement, and a sense of purpose (22), factors that may enable them to better capitalize on serotonergic advantages. Conversely, unemployed patients might experience sustained stress or lack daily structure, potentially limiting the degree to which serotonergic factors relate to early improvement. Notably, this employment-dependent serotonin interaction was limited to 12 weeks, suggesting initial biological advantages become less critical over time as continued treatment and adaptive processes allow others to catch up (23).

At 12 months, lower TNF-α levels were significantly associated with remission exclusively among unemployed participants, suggesting a distinct interplay between immune mechanisms and chronic psychosocial adversity. TNF-α, a pro-inflammatory cytokine, contributes to depression severity and treatment resistance, especially under chronic stress conditions (24). Unemployment typically entails prolonged stress, isolation, and economic hardship—factors associated with elevated inflammatory markers (25). Thus, unemployed individuals with lower baseline TNF-α may experience reduced inflammatory burdens, facilitating sustained recovery. This association was evident only at the 12-month point, likely reflecting the cumulative neuroinflammatory impact of chronic stress over extended periods, underscoring the need for further investigation of inflammation’s role in prolonged psychosocial adversity.

Conversely, at 12 months, lower leptin levels were associated with remission solely among employed patients, indicating context-dependent biomarker effects. Leptin is involved in immunometabolic regulation and energy balance, acting as a pro-inflammatory adipokine linked to depression severity (26, 27). Lower leptin levels may thus reflect reduced inflammation and healthier energy metabolism, conditions potentially promoted by structured employment routines, including physical activity and dietary regulation. Unemployed individuals often exhibit more sedentary behaviors (28), potentially reducing leptin’s prognostic relevance. The delayed emergence of this interaction at 12 months suggests that sustained behavioral and physiological changes contribute substantially to longer-term remission, highlighting leptin’s role beyond initial pharmacological treatment responses.

Additionally, higher BDNF levels at baseline were associated with remission exclusively among unemployed participants at 12 months, highlighting the neurotrophic factor’s importance under conditions of chronic psychosocial stress. BDNF promotes neuroplasticity and synaptic adaptation critical for depression recovery (29). Prolonged unemployment-related stress can suppress BDNF expression (30), potentially compromising neuroplasticity and recovery potential. Unemployed individuals with relatively higher baseline BDNF levels might possess greater neurobiological resilience, which may be related to more favorable long-term outcomes. This association appeared only at 12 months, aligning with the gradual neuroplastic changes induced by antidepressant treatment over extended periods.

No significant employment-dependent interactions emerged for other immune markers (hsCRP, IL-1β, IL-6, IL-4, IL-10) or additional biomarkers (ghrelin, cholesterol, cortisol, folate, homocysteine). Biological redundancy, uniform modulation by antidepressants regardless of employment context, or insufficient baseline variability may explain this lack of findings. Given the scarcity of research on employment-dependent biomarker associations, future studies are essential to clarify whether socioeconomic contexts modulate biomarker-treatment relationships.

Several limitations must be acknowledged. The naturalistic, flexible treatment protocol precluded detailed analyses of individual antidepressant effects or specific medication combinations, due to increased risk of Type I errors from multiple comparisons. Given the large number of comparisons, the potential for false-positive findings is increased, and the observed associations should therefore be interpreted as preliminary signals rather than definitive effects. Accordingly, the absence of multiplicity correction reflects the exploratory intent of the study rather than an inferential conclusion, and the findings are not intended to support confirmatory statistical claims. The use of median splits for biomarker categorization is another limitation, as this approach may reduce variability and obscure more refined dose–response patterns; however, analyses using biomarkers as continuous variables (**Supplementary Table S5**) yielded broadly comparable associations, supporting the robustness of the observed exploratory patterns. Additionally, biomarkers were assessed only at baseline, precluding evaluation of dynamic changes during treatment. Because antidepressant type, dosage, and medication adjustments were individualized within the naturalistic treatment protocol, residual confounding from pharmacotherapy variation cannot be excluded. These factors may influence biomarker levels or treatment trajectories, and future confirmatory studies with standardized medication regimens will be needed to disentangle these effects. Furthermore, our income-based employment classification, though valid and widely used (1, 13), does not fully capture diverse occupational circumstances, such as part-time work, retirement, or homemaking roles. Such misclassification may have diluted or obscured more nuanced employment-related differences, particularly if these subgroups differ systematically in stress exposure, social engagement, or economic security. Employment status may also be shaped by the course of depression itself, and some degree of reverse causation cannot be ruled out; thus, employment should not be interpreted solely as a moderator in this observational design. Finally, generalizability is limited by the use of a single-center cohort from South Korea, and cultural, demographic, and healthcare-system differences may influence the applicability of these findings to other settings. Replication in multi-center and international samples will be essential to establish broader external validity.

Strengths include a relatively large sample size compared to previous biomarker studies, enhancing statistical power and generalizability. Participants underwent rigorous evaluation via structured clinical protocols and standardized scales, ensuring consistent data quality. Furthermore, the unique evaluation of employment-dependent biomarker interactions across acute and long-term phases addresses a critical gap in the literature.

In conclusion, employment-dependent associations of baseline serum serotonin, TNF-α, leptin, and BDNF levels with antidepressant remission outcomes at 12 weeks and 12 months were identified, highlighting the differential relevance of biomarkers depending on employment status across recovery phases. These exploratory findings emphasize the importance of socioeconomic factors in depression prognosis and suggest potential clinical value for employment-tailored biomarker-guided treatment strategies. Future research is needed to independently replicate and further elucidate these employment- and phase-specific biomarker interactions.

## Data Availability

The raw data supporting the conclusions of this article will be made available by the authors, without undue reservation.

## Supplementary material

Supplementary Figure 1 Participants recruitment and flow.

Supplementary Table 1 Baseline characteristics by 12-week and 12-month remission status.
