# Supplementary material for: Employment-dependent associations of serum biomarkers with short- and long-term antidepressant treatment outcomes
Source: Front Psychiatry. 2026 Jan 9;16:1662993. doi: 10.3389/fpsyt.2025.1662993 (PMC12827588; doi:10.3389/fpsyt.2025.1662993)
Supplement: Supplementary Figure 1 — Participants recruitment and flow. [file Table1.docx]

Employment-dependent associations of serum biomarkers with short- and long-term antidepressant treatment outcomes

Jae-Min Kim^a#*^, Hee-Ju Kang^a#^, Ju-Wan Kim^a^, Min Jhon^a^, Ju-Yeon Lee^a^, Sung-Wan Kim^a^, Il-Seon Shin^a^

Supplementary Materials

**Figure S1.** Participants recruitment and flow

**Table S1.** Inter-assay coefficients of variation (CV) of serum biomarkers.

**Table S2.** Baseline characteristics by 12-week and 12-month remission status.

**Table S3.** Associations of serum biomarkers (categorized by median split) with 12-week remission, stratified by employment status.

**Table S4.** Associations of serum biomarkers (categorized by median split) with 12-month remission, stratified by employment status.

**Table S5.** Employment-dependent interactions of continuous serum biomarker levels with the likelihood of achieving remission at 12 weeks and 12 months.

**Fig. S1.** Participants recruitment and flow


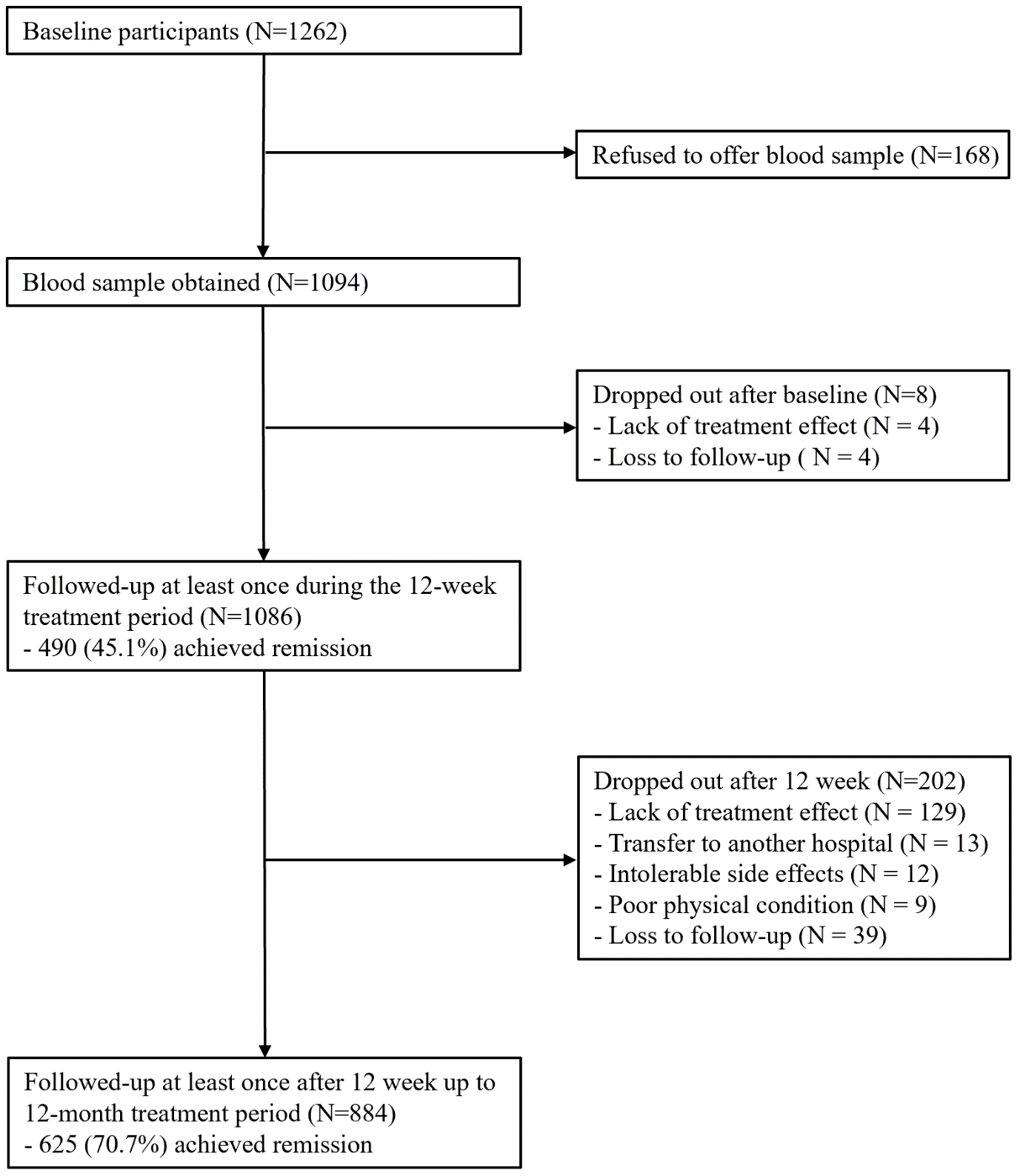


| **Table S1.** Inter-assay coefficients of variation (CV) of serum biomarkers. | | | |
| --- | --- | --- | --- |
|  | Detection Limit | Test times (n) | Mean CV (%) |
| High-sensitivity C-reactive protein, mg/L | 0.03 | 21 | 3.59 |
| Tumor necrosis factor-α, pg/mL | 0.011 | 20 | 6.53 |
| Interleukin-1β, pg/mL | 0.14 | 12 | <15.00 |
| Interleukin-6, pg/mL | 0.11 | 12 | <20.00 |
| Interleukin-4, pg/mL | 1.12 | 12 | <15.00 |
| Interleukin-10, pg/mL | 0.56 | 12 | <20.00 |
| Leptin, ng/mL | 0.2 | 6 | 5.55 |
| Ghrelin, pg/mL | 93 | 30 | 16.3 |
| Total cholesterol, mg/dL | 3.86 | 21 | 1.5 |
| Brain derived neurotrophic factor, ng/mL | 0.02 | 40 | 9.00 |
| Serotonin, ng/mL | 1.0 | 100 | 4.00 |
| Cortisol, μg/dL | 0.054 | 84 | 3.02 |
| Folate, ng/mL | 1.2 | 84 | 5.28 |
| Homocysteine, μmol/L | 1.0 | 80 | 4.32 |

| **Table S2.** Baseline characteristics by 12-week and 12-month remission status. | | | | | | | | |
| --- | --- | --- | --- | --- | --- | --- | --- | --- |
|  | Up to 12-week treatment (N = 1086) | | | | Up to 12-month treatment (N = 884) | | | |
|  | No remission (N = 596) | Remission (N = 490) | Statistical coefficien  ts^a^ | P-  value | No remission (N = 259) | Remission (N = 625) | Statistical coefficien  ts^a^ | P-value |
| **Socio-demographic characteristics** |  |  |  |  |  |  |  |  |
| Age, mean (SD) years | 55.9 (15.6) | 58.2 (13.9) | t=-2.610 | **0.009** | 56.1 (16.0) | 57.3 (14.2) | t=-1.048 | 0.295 |
| Sex, N (%) female | 411 (69.0) | 334 (68.2) | χ^2^=0.079 | 0.778 | 173 (66.8) | 434 (69.4) | χ^2^=0.595 | 0.440 |
| Education, mean (SD) years | 9.1 (4.7) | 9.1 (4.9) | t=+0.065 | 0.948 | 9.3 (4.7) | 9.0 (4.8) | t=+1.090 | 0.276 |
| Marital status, N (%) unmarried | 192 (32.2) | 134 (27.3) | χ^2^=3.033 | 0.082 | 83 (32.0) | 174 (27.8) | χ^2^=1.571 | 0.210 |
| Living alone, N (%) | 94 (15.8) | 73 (14.9) | χ^2^=0.158 | 0.691 | 43 (16.6) | 88 (14.1) | χ^2^=0.923 | 0.337 |
| Unemployed status, N (%) | 186 (31.2) | 130 (26.5) | χ^2^=2.852 | 0.091 | 82 (31.7) | 160 (25.6) | χ^2^=3.383 | 0.066 |
| Household monthly income, N (%) <2,000 USD | 375 (62.9) | 273 (55.7) | χ^2^=5.801 | **0.016** | 167 (64.5) | 355 (56.8) | χ^2^=4.465 | **0.035** |
| **Clinical characteristics** |  |  |  |  |  |  |  |  |
| Major depressive disorder, N (%) | 510 (85.6) | 415 (84.7) | χ^2^=0.164 | 0.686 | 227 (87.6) | 534 (85.4) | χ^2^=0.743 | 0.389 |
| Melancholic feature, N (%) | 95 (15.9) | 67 (13.7) | χ^2^=1.088 | 0.297 | 45 (17.4) | 96 (15.4) | χ^2^=0.554 | 0.457 |
| Atypical feature, N (%)) | 35 (5.9) | 34 (6.9) | χ^2^=0.514 | 0.473 | 16 (6.2) | 39 (6.2) | χ^2^=0.001 | 0.972 |
| Age at onset, mean (SD) years | 49.4 (17.1) | 52.8 (15.7) | t=-3.366 | **0.001** | 49.3 (17.4) | 51.5 (16.0) | t=-1.790 | 0.074 |
| Duration of illness, mean (SD) years | 6.7 (9.6) | 5.7 (9.0) | t=+1.808 | 0.071 | 7.1 (9.5) | 6.1 (9.3) | t=+1.497 | 0.135 |
| Number of depressive episodes, mean (SD) | 2.7 (5.6) | 1.8 (4.2) | t=+2.929 | **0.003** | 3.1 (6.1) | 1.9 (4.5) | t=+2.863 | **0.004** |
| Duration of present episode, mean (SD) months | 8.3 (12.0) | 6.4 (8.0) | t=+3.128 | **0.002** | 8.3 (12.2) | 7.3 (9.5) | t=+1.138 | 0.256 |
| Family history of depression, N (%) | 82 (13.8) | 76 (15.5) | χ^2^=0.664 | 0.415 | 32 (12.4) | 93 (14.9) | χ^2^=0.961 | 0.327 |
| Number of physical disorders, mean (SD) | 1.6 (1.3) | 1.7 (1.2) | t=-1.212 | 0.226 | 1.7 (1.3) | 1.7 (1.3) | t=+0.021 | 0.983 |
| Body mass index, mean (SD) kg/m^2^ | 23.2 (3.3) | 23.2 (3.0) | t=+0.219 | 0.827 | 23.0 (3.3) | 23.4 (3.1) | t=-1.855 | 0.064 |
| Current smoking, N (%) | 80 (13.4) | 43 (8.8) | χ^2^=5.783 | **0.016** | 35 (13.5) | 61 (9.8) | χ^2^=2.665 | 0.103 |
| **Assessment scales**, mean (SD) scores |  |  |  |  |  |  |  |  |
| Hospital Anxiety & Depression Scale-depression subscale | 14.1 (3.9) | 13.1 (4.0) | t=+4.272 | **<0.001** | 14.3 (3.8) | 13.4 (4.0) | t=+3.092 | **0.002** |
| Hospital Anxiety & Depression Scale-anxiety subscale | 12.2 (4.0) | 11.3 (4.1) | t=+3.666 | **<0.001** | 12.3 (4.0) | 11.6 (4.0) | t=+2.521 | **0.012** |
| Social and Occupational Functional Assessment Scale | 55.0 (7.6) | 57.1 (7.1) | t=-4.785 | **<0.001** | 55.6 (7.0) | 56.3 (7.3) | t=-1.028 | 0.227 |
| Alcohol Use Disorders Identification Test | 5.3 (9.2) | 5.4 (8.4) | t=-0.110 | 0.912 | 6.1 (9.8) | 5.1 (8.7) | t=+1.366 | 0.173 |
| ^a^Independent two sample t-test or χ^2^ tests, as appropriate.  Bold style denotes statistical significance (p-values<0.05). | | | | | | | | |

| **Table S3.** Associations of serum biomarkers (categorized by median split) with 12-week remission, stratified by employment status. | | | | | | | | |
| --- | --- | --- | --- | --- | --- | --- | --- | --- |
|  | Cut-off by  median value | Adjusted OR (95% CI) for remission | | |  | Statistics for employment interaction | |  |
|  |  | All patients  (N=1086) | Employed  (N = 770) | Unemployed  (N = 316) |  | Wald | P-value |  |
| High-sensitivity C-reactive protein | <0.45 mg/dL | 2.00 (1.55-2.58)^‡^ | 2.01 (1.49-2.72)^‡^ | 2.05 (1.26-3.32)^‡^ |  | 0.013 | 0.908 |  |
| Tumor necrosis factor-α | <0.59 pg/mL | 1.34 (1.04-1.73)^*^ | 1.39 (1.03-1.87)^*^ | 1.27 (0.78-2.06) |  | 0.144 | 0.704 |  |
| Interleukin-1β | <1.11 pg/mL | 1.47 (1.14-1.89)^†^ | 1.40 (1.04-1.89)^*^ | 1.77 (1.09-2.87)^*^ |  | 0.137 | 0.711 |  |
| Interleukin-6 | <1.65 pg/mL | 1.36 (1.06-1.74)^*^ | 1.51 (1.12-2.02)^†^ | 1.03 (0.64-1.66) |  | 2.113 | 0.146 |  |
| Interleukin-4 | <36.87 pg/mL | 1.19 (0.92-1.53) | 1.24 (0.92-1.68) | 1.08 (0.67-1.75) |  | 0.476 | 0.490 |  |
| Interleukin-10 | <10.72 pg/mL | 1.14 (0.89-1.45) | 1.12 (0.84-1.50) | 1.13 (0.71-1.81) |  | 0.001 | 0.988 |  |
| Leptin | <5.82 ng/mL | 1.59 (1.18-2.14)^†^ | 1.36 (0.96-1.94) | 2.44 (1.34-4.42)^†^ |  | 0.181 | 0.671 |  |
| Ghrelin | >378.00 pg/mL | 1.23 (0.96-1.58) | 1.22 (0.91-1.64) | 1.25 (0.78-2.00) |  | 0.072 | 0.788 |  |
| Total cholesterol | <178.00 mg/dL | 0.97 (0.75-1.24) | 0.95 (0.70-1.28) | 1.00 (0.62-1.60) |  | 0.003 | 0.954 |  |
| Brain derived neurotrophic factor | >23.31 ng/mL | 1.07 (0.84-1.37) | 1.17 (0.88-1.57) | 0.81 (0.50-1.31) |  | 0.799 | 0.371 |  |
| Serotonin | >72.70 ng/mL | 1.22 (0.95-1.57) | 1.37 (1.02-1.84)^*^ | 0.91 (0.56-1.47) |  | 1.420 | 0.233 |  |
| Cortisol | <10.62 μg/dL | 0.81 (0.63-1.03) | 0.90 (0.67-1.20) | 0.61 (0.37-0.98)^*^ |  | 0.990 | 0.320 |  |
| Folate | >7.45 ng/mL | 1.24 (0.96-1.59) | 1.15 (0.85-1.55) | 1.58 (0.97-2.57) |  | 0.799 | 0.371 |  |
| Homocysteine | <10.92 μmol/L | 0.94 (0.72-1.22) | 0.95 (0.70-1.29) | 0.85 (0.51-1.43) |  | 0.167 | 0.683 |  |
| Each odds ratio represents the comparison of high vs. low biomarker groups based on median cut-offs, shown for all patients and separately for employed and unemployed participants, and adjusted for age, sex, marital status, monthly income, number of depressive episode, number of physical disorder, body mass index, and scores on Hospital Anxiety & Depression Scale-depression subscale and Social and Occupational Functional Assessment Scale.  ^*^P<0.05; ^†^P<0.01; ^‡^P<0.001. | | | | | | | |  |

| **Table S4.** Associations of serum biomarkers (categorized by median split) with 12-month remission, stratified by employment status. | | | | | | | | |
| --- | --- | --- | --- | --- | --- | --- | --- | --- |
|  | Cut-off by  median value | Adjusted OR (95% CI) for remission | | |  | Statistics for employment interaction | |  |
|  |  | All patients  (N=884) | Employed  (N = 642) | Unemployed  (N = 242) |  | Wald | P-value |  |
| High-sensitivity C-reactive protein | <0.45 mg/dL | 2.34 (1.71-3.20)^‡^ | 2.73 (1.86-4.00)^‡^ | 2.05 (1.13-3.73)^*^ |  | 1.642 | 0.200 |  |
| Tumor necrosis factor-α | <0.59 pg/mL | 1.67 (1.23-2.27)^†^ | 1.09 (0.60-1.89) | **2.97 (1.71-4.65)**^†^ |  | **4.923** | **0.027** |  |
| Interleukin-1β | <1.11 pg/mL | 1.33 (0.98-1.81) | 1.47 (1.02-2.12)^*^ | 1.17 (0.65-2.11) |  | 0.449 | 0.503 |  |
| Interleukin-6 | <1.65 pg/mL | 1.11 (0.82-1.49) | 1.20 (0.84-1.72) | 0.85 (0.47-1.53) |  | 0.743 | 0.389 |  |
| Interleukin-4 | <36.87 pg/mL | 1.02 (0.75-1.38) | 0.92 (0.64-1.32) | 1.19 (0.66-2.14) |  | 0.827 | 0.363 |  |
| Interleukin-10 | <10.72 pg/mL | 0.91 (0.68-1.23) | 0.81 (0.57-1.16) | 1.27 (0.72-2.27) |  | 1.096 | 0.295 |  |
| Leptin | <5.82 ng/mL | 1.49 (1.04-2.13)^*^ | **1.71 (1.14-2.79)**^*^ | 1.03 (0.48-1.98) |  | **5.183** | **0.023** |  |
| Ghrelin | >378.00 pg/mL | 1.16 (0.86-1.56) | 1.05 (0.74-1.50) | 1.45 (0.80-2.60) |  | 0.734 | 0.392 |  |
| Total cholesterol | <178.00 mg/dL | 1.04 (0.77-1.40) | 1.10 (0.76-1.58) | 0.81 (0.45-1.45) |  | 1.690 | 0.194 |  |
| Brain derived neurotrophic factor | >23.31 ng/mL | 1.36 (1.01-1.84)^*^ | 1.08 (0.72-1.62) | **2.37 (1.39-3.96)**^†^ |  | **4.410** | **0.039** |  |
| Serotonin | >72.70 ng/mL | 1.18 (0.87-1.60) | 1.19 (0.83-1.71) | 1.09 (0.69-1.96) |  | 0.211 | 0.646 |  |
| Cortisol | <10.62 μg/dL | 0.88 (0.65-1.19) | 0.97 (0.68-1.39) | 0.72 (0.41-1.29) |  | 0.257 | 0.612 |  |
| Folate | >7.45 ng/mL | 1.06 (0.78-1.44) | 1.16 (0.80-1.67) | 0.88 (0.48-1.62) |  | 0.031 | 0.860 |  |
| Homocysteine | <10.92 μmol/L | 0.82 (0.49-1.12) | 0.74 (0.51-1.08) | 0.96 (0.50-1.85) |  | 0.772 | 0.380 |  |
| Each odds ratio represents the comparison of high vs. low biomarker groups based on median cut-offs, shown for all patients and separately for employed and unemployed participants, and adjusted age, sex, marital status, monthly income, number of depressive episode, number of physical disorder, body mass index, and scores on Hospital Anxiety & Depression Scale-depression subscale and Social and Occupational Functional Assessment Scale.  ^*^P<0.05; ^†^P<0.01; ^‡^P<0.001. Bold style denotes statistical significance for employment interaction (p-values<0.05). | | | | | | | |  |

| **Table S5.** Employment-dependent interactions of continuous serum biomarker levels with the likelihood of achieving remission at 12 weeks and 12 months. | | | | | |
| --- | --- | --- | --- | --- | --- |
|  | 12-week remission  (N=1086) | |  | 12-month remission  (N=884) | |
|  | Wald | P-value |  | Wald | P-value |
| High-sensitivity C-reactive protein | 0.752 | 0.386 |  | 3.047 | 0.081 |
| Tumor necrosis factor-α | 0.036 | 0.850 |  | **5.354** | **0.021** |
| Interleukin-1β | 1.882 | 0.170 |  | 0.561 | 0.454 |
| Interleukin-6 | 0.217 | 0.641 |  | 0.037 | 0.848 |
| Interleukin-4 | 1.336 | 0.243 |  | 1.785 | 0.181 |
| Interleukin-10 | 1.806 | 0.179 |  | 0.259 | 0.611 |
| Leptin | 0.050 | 0.823 |  | **5.807** | **0.016** |
| Ghrelin | 0.126 | 0.723 |  | 0.696 | 0.404 |
| Total cholesterol | 2.639 | 0.104 |  | 3.048 | 0.081 |
| Brain derived neurotrophic factor | 1.405 | 0.236 |  | **4.442** | **0.038** |
| Serotonin | **4.554** | **0.036** |  | 0.010 | 0.921 |
| Cortisol | 0.053 | 0.817 |  | 0.009 | 0.922 |
| Folate | 0.745 | 0.388 |  | 0.051 | 0.822 |
| Homocysteine | 0.400 | 0.527 |  | 2.567 | 0.109 |
| ^b^Adjustment for age, sex, marital status, monthly income, number of depressive episode, number of physical disorder, body mass index, and scores on Hospital Anxiety & Depression Scale-depression subscale and Social and Occupational Functional Assessment Scale.  Bold style denotes statistical significance (p-values<0.05). | | | | | |
